# Customized pachymetric guided epithelial debridement for corneal collagen cross linking

**DOI:** 10.1186/1471-2415-9-10

**Published:** 2009-08-28

**Authors:** George D Kymionis, Vasilios F Diakonis, Efekan Coskunseven, Mirco Jankov, Sonia H Yoo, Ioannis G Pallikaris

**Affiliations:** 1 Bascom Palmer Eye Institute, Miller School of Medicine, Miami University, FL, USA; 2 Institute of Vision & Optics, Department of Medicine, University of Crete, Crete, Greece; 3 World Eye Hospital, Istanbul, Turkey; 4 University of Beogradu, Department of Medicine, Serbia

## Abstract

**Backround:**

We describe a modified method for deepitheliazation prior to corneal cross linking (CXL). The technique may overcome the current corneal pachymetric limitation parameter (over 400 microns) that is necessary for the safety of the procedure without affecting the overall benefits.

**Methods:**

In a series of two patients, with inferior topographic steepening and regional thinning (less than 400 microns corresponding to the area of corneal steepening), CXL after customized epithelial removal was performed.

**Results:**

There were no intra- or postoperative adverse events seen by the nine month follow up examination. Stabilization of the corneal ectasia was observed up to nine months post-costumized pachymetric-guided epithelial removal.

**Conclusion:**

The technique of customized pachymetric-guided epithelial removal is easy to perform and may overcome the limitations of the preoperative corneal pachymetry expanding the application of the procedure in patients with regional corneal thinning.

## Background

Corneal ectatic diseases (such as keratoconus and post-LASIK corneal ectasia) lead to corneal biomechanical instability, resulting in myopia, irregular astigmatism and loss of best spectacle corrected visual acuity. Recently, corneal cross linking (CXL) has been introduced, a novel technique which increases corneal rigidity, improves corneal biomechanics and stabilizes the progression of corneal ectasia [1-6].

Based on the current protocol in performing CXL, the cross linking effect is limited to the anterior 300 microns of the corneal stroma [7,8]. Both corneal thickness (over 400 microns) and the presence of riboflavin are of critical importance to the process since they prevent UVA from reaching the deep stroma, the endothelium and crystalline lens.

The downside of the corneal pachymetry limitation is that patients with keratoconus or keratectasia often have thinner corneas especially near the apex-cone areas. In order to overcome this limitation, we performed CXL treatment in two patients after customized (according to pachymetric measurements) deepitheliazation (preserving the epithelium in thinner corneal regions where pachymetry was inadequate-less than 400 microns) and we investigated the effectiveness and safety of CXL treatment after this approach.

## Methods

Two patients (one with keratoconus and the other with post LASIK corneal ectasia) with progressive keratectasia (detected by progressive topographic steepening between six months consecutive measuerements) and corneal pachymetry under 400 microns at the area of topographic steepening (380 and 375 microns determined by ultrasound pachymetry) underwent CXL treatment after customized epithelial removal. Central corneal pachymetry were 440 and 435 microns respectively.

**Figure 1:**
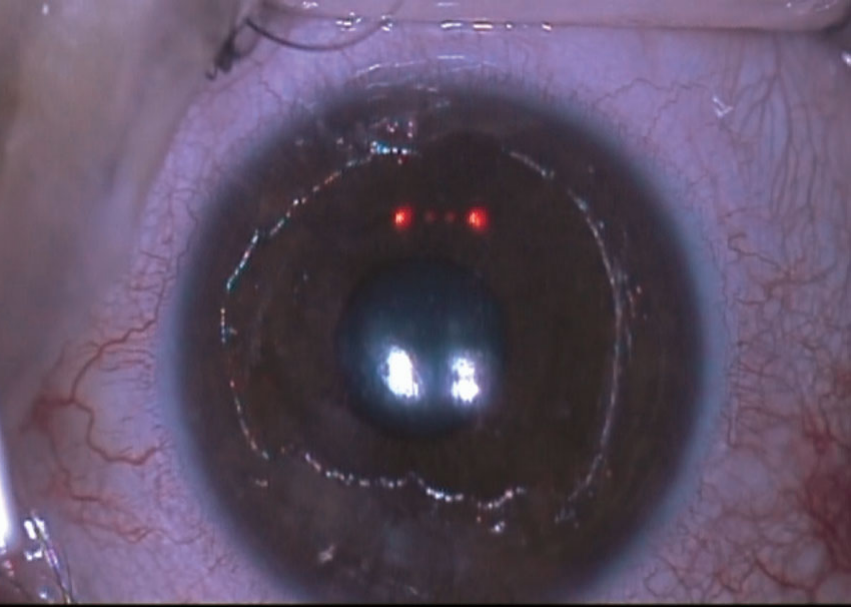
**Intraoperative pachymetric-guided corneal epithelium removal during corneal collagen cross linking in patient with post-LASIK corneal ectasia and inferior corneal thinning**.

**Figure 2:**
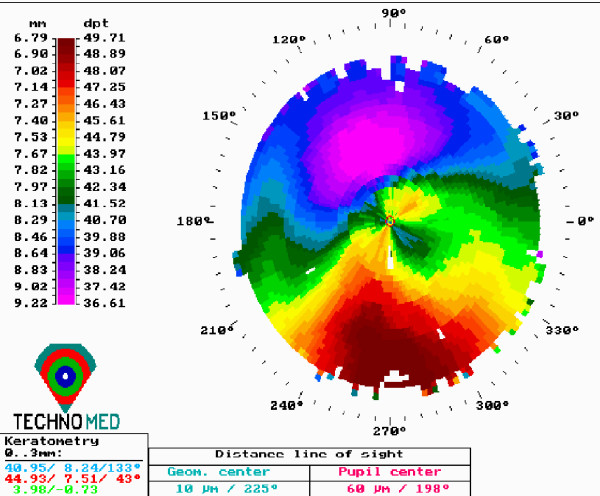
**Preoperative corneal topography in a patient scheduled for customized pachymetric guided epithelial debridement for corneal collagen cross linking**.

The surgical procedure was conducted under sterile conditions. The patient's eye was anesthetized with proparacaine 0.5% (Alcaine). A 8.0 mm diameter of corneal epithelium was mechanically removed using a rotating brush leaving a small localized inferior area of corneal epithelium corresponding to the thinner-area (area of topographic steepening) (Figure 1 and 2a). De-epithelization was followed by instillation of riboflavin (0.1% solution 10 mg riboflavin-5-phosphate) for every 3 minutes for 15 minutes until the stroma was completely penetrated and aqueous was stained yellow. After confirming presence of riboflavin in the corneal tissue and anterior chamber (slit lamp biomicroscopy), UVA irradiation was applied. The UVA irradiation was performed using an optical system (UV-X illumination system version 1000, UVXTM, IROC AG, Zurich, Switzerland), with a light source consisting of an array of UV diodes (365 nm) in conjunction with a potentiometer to allow regulation of voltage. Before treatment, an intended 3.0 mW/cm^2 ^of surface irradiance (5.4 J/cm^2 ^surface dose) was calibrated using a UV light meter at a working distance of 5 cm. Irradiance was performed for 30 minutes at an 8 mm treatment zone. During treatment, riboflavin solution was applied every 4 minutes to ensure saturation and balanced salt solution (BSS) was used to maintain corneal stromal hydration (every 3-5 minutes).

At the end of the procedure, a combination steroid and antibiotic drop (Tobradex, 4 times daily) was administered in all patients and a bandage soft contact lens was kept in place until full corneal reepithelialization occurred.

## Results

**Figure 3:**
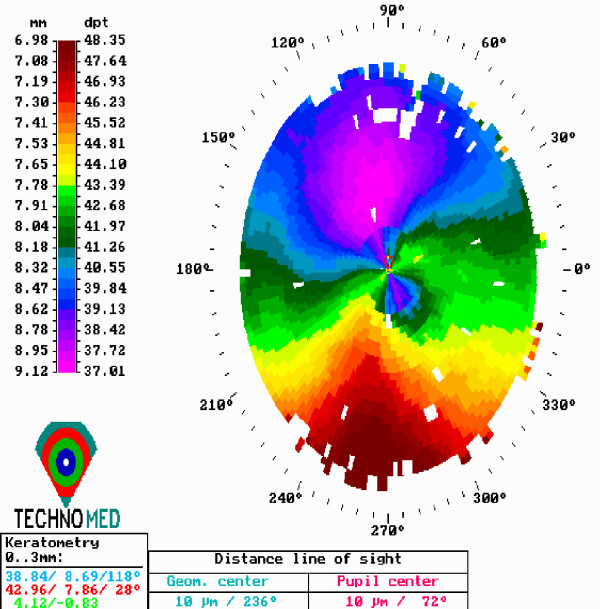
**Last follow up examination corneal topography after customized pachymetric guided epithelial debridement for corneal collagen cross linking (from the same patient as in figure 2)**.

Both procedures were uneventful and no postoperative complications were demonstrated. The epithelium healed completely 5 days after the surgery, and the bandage contact lenses were removed. Nine months after the procedures, corneal topography in both patients showed stability (Figures 2 and 3), while confocal microscopy analysis demonstrated no endothelial cell density alterations (preoperative were 2512 ± 42 and 2725 ± 102, to 2502 ± 55 and 2738 ± 58 postoperative, respectively).

## Discussion

Corneal cross linking with the use of photosynthesizing riboflavin and UVA has demonstrated satisfactory results in stabilizing keratoconic and keratectatic corneas. A major limitation of the technique is that its application is contraindicated in corneas with stromal pachymetry less than 400 micrometers. This is due to the fact that an irradiance of 0.37 mW/cm^2 ^has been found to be cytotoxic for the endothelial cell layer. Since the absorption coefficient for the human corneas is 70 cm^-1 ^and the intended surface irradiance is 3.0 mW/cm^2^, the 0.37 mW/cm^2^irradiance is reached at 300 microns depth. In a 400 microns thick cornea saturated with riboflavin, the irradiance at the endothelial level is 0.18 mW/cm^2^, which is a factor of 2 smaller than the damage threshold. Therefore, the 400 microns limit is considered the safe limit to protect the endothelium and intraocular structures from the adverse effects of UV irradiation and it has been established as a clinical standard [6-8]. Unfortunately, the very patients who are in need of CXL, those with ectasia, are the same ones who have thin corneas often below the threshold of that considered to be safe for CXL treatment.

In order to avoid this limitation, we performed CXL treatment in two patients (thinnest pachymetry of 380 and 375, respectively) after pachymetric-guided corneal epithelial removal, leaving a small localized inferior area of corneal epithelium corresponding to the thinnest area over the apex of the cone. The procedure was performed uneventfully while no adverse effects were found during the nine month follow up period.

In a recent publication, Hafezi et al [5] report a case of localized endothelial damage with transient stromal edema (0.75 mm × 0.75 mm) in the central deep cornea exactly below the corneal apex, corresponding to the area of the greatest steepness and corneal thinning. The stromal edema slowly resolved during the following 6 weeks. The authors suggest increasing stromal thickness to 400 microns by avoiding dehydrating the cornea using an alternative riboflavin solution without dextran after epithelium debridement. In the technique described in the current manuscript, we not only prevent local stromal dehydration by maintaining the epithelium and by using BSS, but we also, by the preservation of the corneal epithelium, may blocked the excessive UV irradiation in this vulnerable area, increasing the safety of the procedure.

During the CXL procedure, the epithelium is removed and riboflavin solution is placed onto the cornea until it penetrates into the anterior chamber and then UV irradiation is applied. BSS was used in the current study to maintain corneal stromal hydration (the current protocol suggests the use of hypoosolar riboflavin) in between application of riboflavin solution taking care not to over-dilute the riboflavin solution which is necessary for cross linking and protection of the intraocular structures (eg, lens). Since the cone is denuded of epithelium, it is vulnerable to excessive dehydration during the lengthy CXL treatment (total of 45 minutes). The selective preservation of epithelium in our technique may help to minimize the risk of excess dehydration and inadvertent local damage to the endothelium in the thinnest areas of the cornea.

## Conclusion

In conclusion, localized pachymetric-guided epithelial removal could increase the safety of the procedure with localized low pachymetry (under 400 microns) without adversely affecting the final results of corneal cross linking. Some limitations of this technique are the long term efficacy of the modified technique, the ability of the epithelium to absorb the UVA radiation (epithelium preferentially absorbs UVB and UVC over UVA radiation) [9], alterations in riboflavin corneal stromal saturation and the possibility of partially inadequate treatment in the areas covered by epithelium. Future comparative studies should be performed in order to elucidate these limitations.

## Competing interests

The authors have no financial or proprietary interest in any materials or methods described herein and they have equally contributed for the preparation of this manuscript.

GDK has received for the academic year 2009 a scholarship of 15.000 Euro from the 'Hellenic society of intraocular implants and refractive surgery' as an economic aid for a fellowship program.

## Authors' contributions

All authors have read the final version of the manuscript and approve it for publication.

## Pre-publication history

The pre-publication history for this paper can be accessed here:


